# Prevalence of prescribing in pregnancy using the Irish primary care research network: a pilot study

**DOI:** 10.1186/s12884-015-0489-0

**Published:** 2015-03-26

**Authors:** Paul Dillon, Kirsty K O’Brien, Ronan McDonnell, Erica Donnelly-Swift, Rose Galvin, Adam Roche, Kate Cronin, David R Walsh, Rowan Schelten, Susan Smith, Tom Fahey

**Affiliations:** HRB Centre for Primary Care Research, Department of General Practice, Royal College of Surgeons in Ireland, 123 St Stephen’s Green, Dublin, 2 Ireland

**Keywords:** Prescribing, Medication use, Pregnancy, FDA pregnancy-risk categories

## Abstract

**Background:**

To establish the prevalence and patterns of prescribing to pregnant women in an Irish primary care setting.

**Methods:**

We reviewed electronic healthcare records routinely collected in primary care, of pregnant women attending nine Dublin-based General Practices affiliated to the Irish Primary Care Research Network (IPCRN) for antenatal care between January 2007 and October 2013 (n = 2,361 pregnancies).

**Results:**

Excluding folic acid, 46.8% (n = 1,104) of pregnant women were prescribed at least one medication. Amoxicillin (11.1%, n = 263) and co-amoxiclav (8.0%, n = 190) were the most commonly prescribed medication followed by topical clotrimazole (4.9%, n = 117), salbutamol inhalers (4.1%, n = 96) and paracetamol (4.0%, n = 95). General Medical Services (GMS) patients were more likely to receive a prescription than private patients (OR 2.81; 95%CI (2.28, 3.47)). We applied the US FDA pregnancy-risk categories as a proxy measure of prescribing appropriateness, with FDA Category D and X medications considered inappropriate. FDA Category D drugs were prescribed in 5.9% (n = 140) of pregnancies. FDA Category X drugs were prescribed in 4.9% (n = 116) of pregnancies but after exclusion of oral contraceptives, progestogens, infertility treatments Category X medications were prescribed in 0.6% (n = 13) of pregnancies. After the initial antenatal consultation the prescribing prevalence of FDA Category D medications reduced to 4.7% (n = 110) and Category X to 3.1% (n = 72).

**Conclusions:**

The overall prevalence of prescribing to pregnant women in our cohort is low compared to studies internationally, however similar levels of prescribing for FDA Category D and X were found. Following the initial antenatal consultation levels of prescribing of the FDA Category D and X medications reduced, however there is potential to further reduce their use in early pregnancy. The IPCRN database has provided valuable information on the current practice of antenatal prescribing within this pilot group of practices however it is limited by the absence of morbidity and pregnancy outcome data.

## Background

The use of medication in pregnancy is often necessary for the treatment of acute or chronic illnesses. Poor management of maternal diseases can have a negative effect on both the health of the mother and the foetus and thus medication use is often beneficial. Medication use may also be inadvertent. It has been reported that up to half of pregnancies are unplanned [1] and medication may be prescribed before the general practitioner (GP) or the patient is aware of the pregnancy.

The prevalence of medication use in pregnancy has been widely reported, however international estimates vary from 40-99% [2-8]. Variability in the research methodologies and also in the types of medication used between countries may contribute to the differences in reported prevalence [9,10]. Observational research however has demonstrated that certain medications have the potential to cause adverse effects to the foetus including anatomical malformations, impaired physiological functions, alterations to growth, and foetal and infant mortality but also delayed subtle effects on social and intellectual function [11]. The US Food and Drug Administration (FDA) developed a classification system (Table 1) to categorise drugs based on foetal-harm risk. The classification is based on whether there is evidence of harm in human or animal studies. The majority of drugs have been classified as category C [12], which indicates a lack of robust studies assessing human foetal harm, demonstrating the lack of safety data for the use of many drugs in pregnancy. Despite this uncertainty, studies have reported high prevalence (10-80%) of use of category C drugs [4,6,8]. For category D and category X drugs (positive evidence of foetal harm), studies from various countries such as the US, the UK, France and Ireland have reported the use of these medicines by 2.5-59.3% and 0.6-4.6% of pregnant women, respectively [2,4,6,8,13].

**Table 1:** **US Food and Drug Administration Category Definitions**

	**Definition**
**Category A**	Controlled studies in women fail to demonstrate a risk to the fetus in the 1st trimester (and there is no evidence of a risk in later trimesters), and the possibility of fetal harm appears remote.
**Category B**	Either animal-reproduction studies have not demonstrated a fetal risk but there are no controlled studies in pregnant women or animal-reproduction studies have shown an adverse effect (other than a decrease in fertility) that was not confirmed in controlled studies in women in the 1st trimester (and there is no evidence of a risk in later trimesters).
**Category C**	Either studies in animals have revealed adverse effects on the fetus (teratogenic or embryocidal or other) and there are no controlled studies in women or studies in women and animals are not available. Drugs should be given only if the potential benefit justifies the potential risk to the fetus.
**Category D**	There is positive evidence of human fetal risk, but the benefits from use in pregnant women may be acceptable despite the risk (e.g., if the drug is needed in a life-threatening situation or for a serious disease for which safer drugs cannot be used or are ineffective).
**Category X**	Studies in animals or human beings have demonstrated fetal abnormalities or there is evidence of fetal risk based on human experience or both, and the risk of the use of the drug in pregnant women clearly outweighs any possible benefit. The drug is contraindicated in women who are or may become pregnant.

Shortcomings in the clarity of the pregnancy-risk classifications have led the FDA to initiate a change to the medicinal-product labelling rules in the US [14]. The FDA categories are often mistakenly viewed as a grading classification with increasing risk from A to X and do not inform on potential harm to the mother from withholding treatment [15]. Similar classification systems exist in Australia and Sweden, however discrepancies between the three classifications for the same drug bring into question the usefulness of these systems [16]. The FDA will soon replace the categories with new pregnancy risk summary, clinical considerations and data sections, for both patients and clinicians. They aim to enable prescribers improve benefit-risk assessments of medication use in pregnancy [17,18].

This is the first study to examine the prevalence of prescribing to pregnant women in an Irish primary care setting using the recently established Irish Primary Care Research Network (IPCRN). Additionally few studies have examined the prevalence of prescribing throughout pregnancy using routine data collected in a primary care setting. Therefore, the overall aim of this study is to establish the prevalence, patterns and appropriateness of prescribing during pregnancy in a pilot group of GP practices affiliated to the Irish Primary Care Research Network (IPCRN). In the absence of explicit criteria we have applied the US FDA pregnancy-risk categories as a proxy measure of prescribing appropriateness in pregnancy. The objectives of the study are to 1) establish the prevalence of prescribing to pregnant women in these practices using the IPCRN’s Maternity Safe Prescribing tool and 2) classify the prescribed medications by the World Health Organisation’s Anatomical Therapeutic Chemical (ATC) codes and by FDA pregnancy-risk category.

## Methods

Ethics approval for this study was granted by the research ethics committee of the Royal College of Surgeons in Ireland. Ethics was obtained for the study and informed consent was received from the data controller (the GP).

### Study design and setting

This was a retrospective cohort study of pregnant women attending nine IPCRN-affiliated GP practices located in the Dublin area. The IPCRN is a national network of GP practices created through collaboration between the Irish College of General Practitioners (ICGP), National University of Ireland, Galway (NUIG), and the Royal College of Surgeons in Ireland (RCSI). It facilitates participating GPs in the areas of research and audit, enabling them to become involved in planned research trials and providing them with new software applications to audit and manage their patient care. Through the IPCRN anonymised GP practice data can be extracted for research purposes. Permission to use the anonymised electronic health records was obtained from the GP and in agreement with the IPCRN.

The Irish health care system consists of mixed public and private funding. Eligibility for primary-care services consist of General Medical Scheme (GMS) card holders who are entitled to free medical visits and pay a small levy for dispensed medications, doctor visit card (DVC) holders who are entitled to free GP visits but must pay for prescriptions in full, and private patients (PRV) who pay for the GP visit and the subsequent dispensed medications. Eligibility for GMS and DVC cards are by means-testing based on income [19,20]. All pregnant women in Ireland however are entitled to a limited number of free antenatal consultations with the GP and hospital obstetrician or midwife [21].

### Participants

An IPCRN software tool identified the patients of interest from the electronically stored medical records of each GP practice and extracted and uploaded anonymised patient information to a secure IPCRN server. All female patients who attended these practices for antenatal care between January 2007 and October 2013 were eligible for inclusion. Patients were excluded if their date of birth was missing, or if their date of birth preceded 1960 or if it followed 1996. Patients were also excluded in cases where both the last menstrual period (LMP) and estimated date of delivery (EDD) were absent, or where they equalled each other, or where the recorded date of the initial antenatal consultation did not occur between the LMP and EDD. These records may have been entered in error or may have been used for training purposes by the GP. Patients whose LMP proceeded the study start date (January 2007) or whose EDD followed the study end-date (October 2013) were excluded, as full prescription records for the entire antenatal period would not be available. Pregnancies recorded as ending in miscarriage were excluded. Duplicate pregnancy records were also identified and removed.

### Data sources and variables

The tool extracted data including year of birth of mother, patient-payment status (GMS, DVC or PRV), LMP, EDD, date of initial antenatal consultation and medication prescribed (including date of issue and duration of therapy). The IPCRN tool also generated a maternity safe prescribing report for each GP practice including information on the most commonly prescribed medications within the practice and any associated pregnancy risk warnings.

### Data verification

To ensure the quality and rigour of the IPCRN software tools, researchers attended the GP practices to confirm that the data extracted by the tool mapped the correct patients attending for antenatal care and their related prescribed medication. The anonymised collated dataset was also verified by researchers attending the GP practices to ensure accuracy following data manipulation.

### Data extraction

Anonymised information on patients’ demographics, pregnancy details, and prescriptions were extracted. Medicines were considered prescribed during the antenatal period if the date of issue of the prescription fell between the LMP and EDD. Prescriptions were labelled with trimester of issue (defined as three 90 day periods from the LMP) and whether it was issued prior to the initial antenatal consultation date. A medicinal product dictionary was created linking the prescribed brand name to generic name, ATC code and FDA pregnancy-risk category (if available). ATC codes were assigned using the Irish Pharmacy Union’s monthly drug formulary download (May 2014) [22]. ‘Briggs: Drugs in Pregnancy and Lactation (8th edition)’ was used to identify FDA categories [11]. Products containing a combination of active substances were classified according to the most stringent FDA category of the concerned active substances. Medications that have not been marketed or approved in the US are unclassified. Trimester of use of medication was considered in cases where the FDA category was dependant on this (e.g. diclofenac is category B but changes classification to category D if used in 3rd trimester or near delivery).

### Statistical analysis

Descriptive statistics were used to characterise the patient population including age (from year of birth to year of antenatal consultation), patient payment status (GMS, DVC or PRV) and prevalence of miscarriage. Descriptive statistics were also used to characterise medications prescribed in pregnancy. The prevalence of prescribing of any medication during the antenatal period was calculated excluding Folic Acid. Prevalence of prescribing after the initial antenatal consultation was also determined. An additional measure of prevalence excluding all medications that are also available over-the-counter is reported. The most commonly prescribed medications are reported, with each medication counted once per pregnancy in cases of multiple/repeat prescriptions.

Prescribing prevalence was calculated by patient payment status. PRV and DVC patients have been grouped on the basis that they receive non-reimbursable prescriptions. Prescribed medications were categorised per level one ATC group, an indicator of the nature of prescribing. The FDA categories can be used to determine the appropriateness of prescribing during pregnancy and the percentage of pregnancies exposed per FDA category is reported. Category D and X medications in this report have been considered potentially harmful and the most commonly prescribed medications from these categories have been reported. Oral contraceptives, fertility treatments and progestogens are category D/X medications, but are not considered to carry the same risk as other drugs in these categories [23,24]. These have been classified separately. Oxytocin is a category X medication used in the induction of labour and has also been classified separately. The prevalence of prescribing of category D and X medication after the initial antenatal consultation has also been determined.

Logistic regression models were used to model binary outcome of prescribing during pregnancy and inappropriate prescriptions (category D and X medications, excluding oral contraceptives, fertility treatments, progestogens and oxytocin) during pregnancy. Univariate and multivariate logistic regression analysis were used to assess combinations of variables. Likelihood ratio (L-R) tests and Wald tests were used to validate variable selection for the model. Model results are presented as odds ratios (OR) and their corresponding 95% confidence intervals (95% CI). The model variables were patient age and GMS or PRV/DVC status.

All statistical analysis was performed using Stata version 13 (StataCorp College Station, Texas, USA).

## Results

### Participants

Data on 2696 patients, representing 3505 pregnancies were obtained from nine Dublin GP practices affiliated with the IPCRN. This represented all consultations for antenatal care in these practices between January 2007 and October 2013.

**Figure 1:**
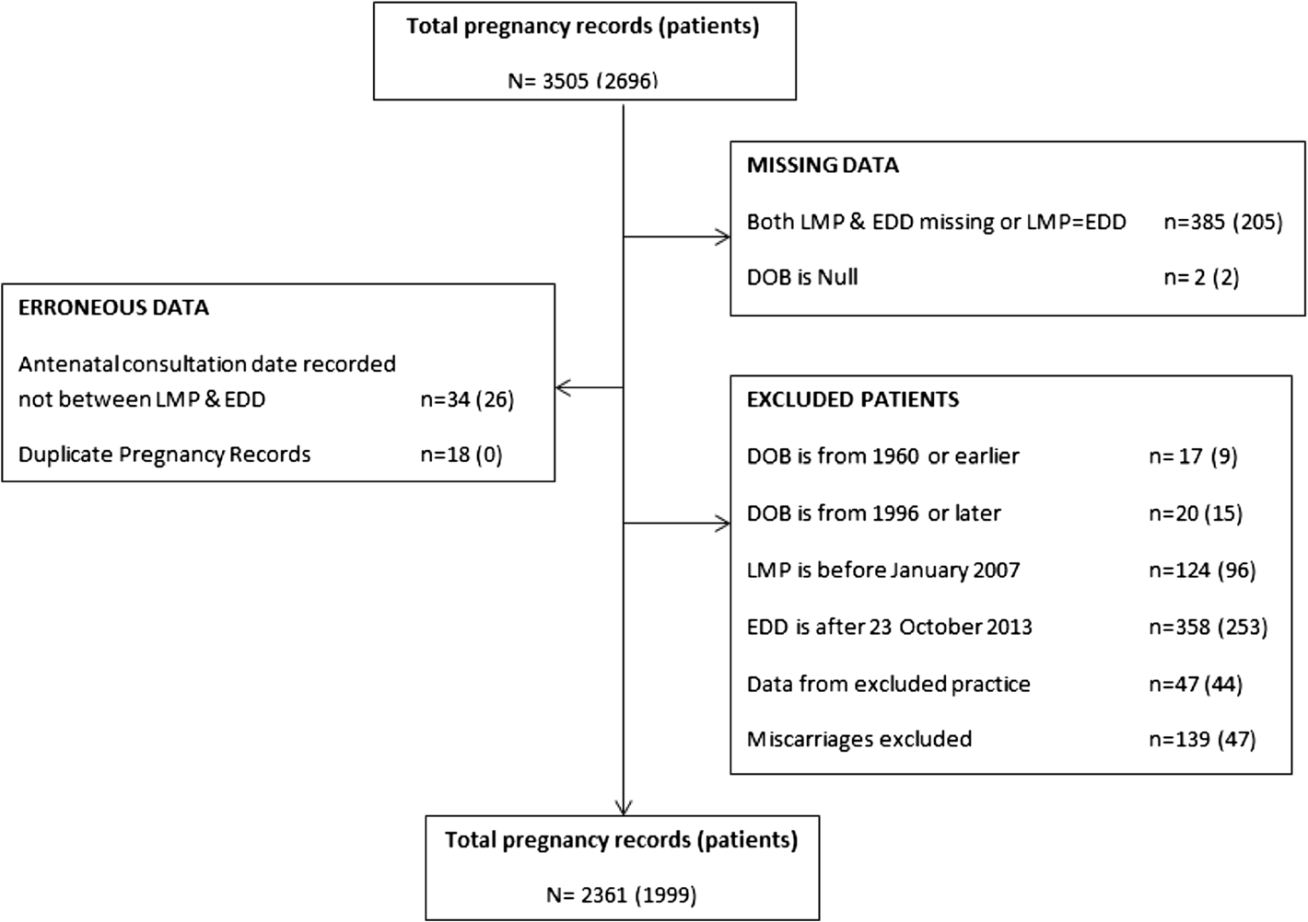
**Flowchart of entry of patients into study and exclusion from study.** Note: A pregnancy (patient) may be excluded under multiple exclusion criteria, however the exclusion criteria were applied sequentially to the dataset, and thus an excluded pregnancy (patient) is only represented once in the flowchart.

Data from one practice were subsequently excluded as it was apparent that prescriptions issued privately (DVC and PRV patients) from this practice were not recorded in the electronic medical records from which the prescribing data was extracted. Following the application of our inclusion criteria, a total of 2361 valid pregnancy records, representing 1999 women from eight GP practices, were included in the analysis. Figure 1 describes the flow of patients through the study.

The average age of participants at time of pregnancy was 31.6 years (range 16–48 years). The majority of women in our study were private patients (n = 1476, 73.8%), 474 (23.7%) held a GMS card and 39 women (2.0%) were DVC holders. Data were missing for 10 patients.

Miscarriage was recorded in 139 pregnancies (5.6%). The range between practices varied from 1.9%-12.5%. Data on miscarriage were missing for 41 (1.6%) of pregnancies.

### Prevalence and prescribing patterns during pregnancy

Excluding folic acid 46.8% (n = 1104) of pregnancies were prescribed at least one medication. Following the initial consultation for antenatal care 43.9% (n = 1036) of pregnancies received a prescription. Excluding any medication that is available over-the-counter, 40.1% (n = 947) of pregnancies were prescribed medication.

Table 2 details the most commonly prescribed medications overall and by trimester (excluding medication that is available over-the-counter). The most commonly prescribed medication were amoxicillin and co-amoxiclav (amoxicillin/clavulanic acid) for treatment of bacterial infections. This was followed by salbutamol inhalers for asthma and, oral contraceptives and beclomethasone inhalers. The most commonly prescribed medications also available over-the-counter were clotrimazole (topical creams and pessaries) for treatment of candida infections and paracetamol.

**Table 2:** **Most commonly prescribed medications during pregnancy (excluding medication available over-the-counter) – the percentage of pregnancies exposed by trimester and overall**

	**Overall (%)**	**1st trimester (%)**	**2nd trimester (%)**	**3rd trimester (%)**
**Amoxicillin**	11.1	3.6	4.8	4.0
**Co-Amoxiclav**	8.0	2.3	2.6	3.8
**Salbutamol**	4.1	1.4	1.7	2.2
**Oral Contraceptives**	3.3	2.4	0.9	1.2
**Beclometasone**	2.1	0.6	1.1	0.9

Note: for the overall column each medication is only counted once per pregnancy in cases of repeat or multiple prescriptions that have been issued in different trimesters.

Table 3 displays the prevalence of prescribing firstly excluding folic acid and secondly excluding medication also available over the counter by patient type. Excluding medication available over-the-counter and adjusting for model factors, GMS patients (OR 2.81; 95% CI (2.28, 3.47)) were more likely to have a prescription issued during pregnancy than PRV/DVC patients. Table 4 details the factors associated with prescribing during pregnancy.

**Table 3:** **Prevalence of prescribing excluding folic acid and prevalence of prescribing excluding medication available over-the-counter by patient-payment type (GMS or PRV/DVC) per pregnancy**

	**Number of valid pregnancies**	**Percentage of pregnancies with a prescription (excluding folic acid) (%)**	**Percentage of pregnancies with a prescription (excluding OTC medications) (%)**
**Overall**	2361	46.8	40.1
**GMS patients**	563	69.4	59.1
**PRV and DVC patients**	1786	39.5	34.0

**Table 4:** **Logistic regression results for the factors associated with any prescription (excluding medication available over-the-counter) issued during pregnancy and any inappropriate prescription (category D and X medications only, excluding medication available over-the-counter) issued during pregnancy**

	**Any prescription during pregnancy (excluding OTC medication)**		**Any inappropriate prescription during pregnancy (excluding OTC medication)**	
**Factor**	**Crude OR (95% CI)**	**Adjusted OR (95% CI)**	**Crude OR (95% CI)**	**Adjusted OR (95% CI)**
**Age**				
16-19 years	3.47 (1.85,6.52)	2.04* (1.06,3.90)	2.34 (0.76,7.19)	1.44 (0.46,4.48)
20-24 years	1.88* (1.35,2.63)	1.46* (1.03,2.07)	2.81 (1.47,5.39)	2.18* (1.12,4.22)
25-29 years	1.0	1.0	1.0	1.0
30-34 years	1.13 (0.90,1.42)	1.35* (1.07,1.72)	1.09 (0.62,1.91)	1.32 (0.74,2.35)
35-39 years	1.10 (0.86,1.40)	1.34* (1.04,1.74)	1.65 (0.94,2.91)	2.07* (1.16,3.69)
≥40 years	1.66 (1.13,2.44)	1.97** (1.33,2.93)	2.01 (0.91,4.44)	2.38* (1.07,5.31)
**Patient status**				
PRV/DVC	1.0		1.0	
GMS	3.48 (2.32,3.42)	2.81** (2.28,3.47)	2.71 (2.32,3.90)	2.74** (2.84,4.08)

*p < 0.03, **p < 0.001.

**Figure 2:**
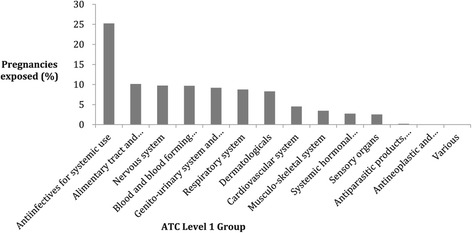
**The percentage of pregnancies exposed by WHO ATC Level 1 Group medication categories.**

Figure 2 details the percentage of pregnancies exposed to at least one drug from each WHO ATC Level 1 group category. Over 25% of pregnancies were prescribed an anti-infective for systemic use such as amoxicillin, co-amoxiclav, and nitrofurantoin. Alimentary tract and metabolism was the next highest Level 1 group category which includes antacids, H2-antagonists, proton-pump inhibitors, laxatives and anti-diabetic medication.

### Appropriateness of prescribing

Category A medications (excluding folic acid, vitamins and minerals) were prescribed to 1.5% (n = 36), category B to 32.6% (n = 766), category C to 21.6% (n = 510), category D to 5.9% (n = 140) and category X to 4.9% (n = 116) of pregnancies. Excluding oral contraceptives, fertility treatments, progestogens and oxytocin, 0.6% (n = 13) of pregnancies were prescribed a category X medication. Table 5 details the most commonly prescribed category D and category X medications and the percentage of pregnancies exposed to each. Following the initial consultation for antenatal care 4.7% (n = 110) and 3.1% (n = 72) were prescribed category D and category X medications (excluding oral contraceptives, fertility treatments, progestogens and oxytocin; category X 0.4%, n = 10). Category D medication prescribed following the initial consultation mainly consisted of anti-depressants, benzodiazepines and non-steroidal anti-inflammatories.

**Table 5:** **Most common FDA category D medication and category X medication prescribed**

**Category D**	**No. of pregnancies exposed**	**Category X**	**No. of pregnancies exposed**
Diclofenac*	19	Rosuvastatin	4
Mefenamic acid*	16	Flurazepam	3
Diazepam	15	Atorvastatin	2
Alprazolam	11	Temazepam	2
Prednisolone*	10	Misoprostol	2
Betamethasone*	10	Diclofenac/Misoprostol	1

Note: each medication is only counted once per pregnancy in cases of repeat prescriptions or multiple prescriptions issued during the same pregnancy.

*Diclofenac is classified as Category B in 1st and 2nd trimester and Category D in 3rd trimester. Mefenamic acid is classified as Category C in 1st and 2nd trimester and Category D in 3rd trimester. Prednisolone and betamethasone are classified as Category C in 2nd and 3rd trimester and Category D in 1st trimester.

Adjusting for model factors, inappropriate prescribing during pregnancy was more likely for patients aged 20–24 years (OR 2.18; 95% CI (1.12, 4.22)), 35–39 years (OR 2.07; 95% CI (1.16, 3.69)) and aged over 40 years (OR 2.38; 95% CI (1.07, 5.31)) than patients aged 25-29 years (p < 0.03). GMS patients were more likely to have an inappropriate prescription prescribed during pregnancy than PRV/DVC status patients (OR 2.74; 95% CI (2.84, 4.08); p < 0.001). Table 4 details the results for the factors associated with inappropriate prescribing during pregnancy.

## Discussion

### Key findings

This study used a routine dataset of electronic medical records extracted from a pilot group of nine GP practices in the Dublin area. We found that at least one prescription was issued to almost half of the pregnancies included in the dataset. GMS patients were significantly more likely to be issued a prescription than private patients. The most commonly prescribed medications were systemic anti-infectives (amoxicillin +/− clavulanic acid), salbutamol inhalers, oral contraceptives and beclomethasone inhalers. FDA Category D medications were prescribed in 5.9% of pregnancies and category X medications in 4.9% of pregnancies. Once oral contraceptives, progestogens, infertility treatments have been excluded the percentage of pregnancies prescribed Category X medications reduced to 0.6%.

### Findings in the context of other studies

Various methods including use of pharmacy records, and patient interview have been used to evaluate maternity prescribing prevalence and thus comparing results across studies is difficult. However, a previous Irish study conducted in an out-patient setting examined the prevalence of prescribing in pregnancy by patient interview and self-report [8]. Medication use in early pregnancy (up-to 20 weeks) was assessed and almost 40% reported using a medication other than folic acid. Category D and X medications were reported by 2.4% and 0.1% of women respectively (excluding oral contraceptives, fertility treatments and progestogens). These results are broadly comparable to our findings. The higher overall prevalence found in our study can be attributed to prescription data captured throughout the entire pregnancy, while the higher category D prevalence in our study has been inflated by prescription of diclofenac and mefenamic acid. These are classified as category B and C in early pregnancy but when used in the 3rd trimester are classified as category D. Additionally, in comparison to studies based on self-report of medication use, higher prevalence estimates can be found from prescribing studies as patient adherence to medication is not accounted for and the additional reluctance of pregnant women to take prescribed medication due to fear of potential foetal harm [25].

A UK study similarly examined prescriptions from general practice during pregnancy, found an overall prescribing prevalence of 65% [2]. The prescription data captured however includes folic acid and a shorter period of the pregnancy than our study (90 days prior to and 70 days after the earliest medical record of pregnancy recognition) making it difficult to make comparisons to our study.

Studies examining medication use throughout the duration of pregnancy have also found higher overall prevalence (57-99%) [3-6], higher prevalence of category D medication (3.6-59.3%) but lower prevalence of category X medication (1.6-4.6%) [4,6,13] in comparison to our study. Our reported prevalence of category X drugs (4.9%) however is inflated by hormonal contraceptives and progestogens, which are largely concentrated in trimester 1 (see Table 2). The low overall prevalence of prescribing found in our cohort may be a true finding or the result of differences in data collection with these studies; however differences in prescribing practices and types of medication used between countries may contribute to the observed variation [9]. Furthermore prescriptions issued outside of primary-care (e.g. prescriptions issued directly from hospital obstetricians) and use of over-the-counter medications, have not been captured in our study.

The study by Cleary et al. reported that private patients were more likely to report any medication use [8] which contrasts with our findings that GMS patients were more likely to receive a prescription. Certain reimbursable over-the-counter and supplement products (paracetamol, clotrimazole, ferrous fumarate) were prescribed often to GMS patients in our study, but for which the GP would not prescribe for PRV/DVC patients. However even when these products were excluded, the association remained. In addition PRV/DVC patients receive prescriptions directly from hospital obstetricians, whereas GMS patients, for reimbursement reasons, have hospital prescriptions transcribed by their GP. Another potential confounding factor is the differences in morbidity between the GMS and PRV/DVC populations, which could not be determined due to the absence of morbidity data in the IPCRN dataset.

Reductions in overall prescribing and prescribing of Category D and Category X were observed following the initial antenatal consultation, mirroring the trend of many studies [2,4,13]. In our cohort, category D and X medications prescribed following the initial consultation mainly included anti-depressants and benzodiazepines which may be appropriate in individual circumstances (moderate-to-severe depression, issues of addiction). It appears prescribers evaluate the risk-benefit of medication use in pregnancy during the initial antenatal consultation however there is a high use of potential teratogens in early pregnancy, a time period where there is a clear and greater risk of teratogenic effect [11]. Research has shown that unplanned pregnancy, socioeconomic group, age, maternal chronic disease, multiparous pregnancy, and use of a potential teratogen prior to conception affect the mother’s risk of potential teratogen use in early pregnancy [8,13]. In our study, GMS status (a proxy indicator for low socioeconomic status) was associated with an increased risk of an inappropriate prescription.

### Strengths and limitations

This is the first study to examine the prevalence of prescribing using data from Irish GP records. Previous studies that have investigated medication use during pregnancy have used different methodologies to determine the prevalence, including the use of self-report questionnaires or dispensing records and may only examine a particular period of time, or time-point in pregnancy. This study overcomes the problem of poor recall in self-report studies through the use of electronic prescription records and examines prescription medication use throughout pregnancy. However, our findings need to be interpreted in the context of the study limitations.

Firstly, this was a convenience sample of nine General Practices and the results are not nationally representative. Secondly the validation process of the collated IPCRN dataset identified certain issues. These relate to prescriptions tagged as “cancelled” in the GP’s records and certain repeat prescriptions issued with a long duration of therapy beyond normal practice (maximum usually 6 months). The issues have affected the accuracy of a small number of individual prescription records within the IPCRN dataset that have been validated against the GP’s own records. The issues will be rectified for future data extraction. Thirdly, gestational length is estimated in the IPCRN dataset. At time of initial antenatal visit the GP records the patient’s recall of LMP, and delivery date is estimated by adding 40 weeks. The occurrence of a miscarriage was poorly recorded in the IPCRN dataset. A separate analysis of pregnancies that ended in miscarriage could not be performed as the date of the miscarriage is not recorded. Additionally a small number of pregnancies that ended in miscarriage may have been included in the analysis if they were not recorded by the GP. Finally maternal morbidity data and pregnancy outcome data (stillbirths, congenital anomalies etc.) are not present in the dataset due to the manner in which they are currently recorded in Irish GP databases (low levels of morbidity-coding; hospital delivery letters attached as scans to electronic medical records). Reliable exposure data, outcome data and data such as prescription indication and maternal comorbidity are important aspects of evaluating medication safety in pregnancy. In many healthcare databases outcome data are limited and exposure data from prescription or dispensing registries are often linked to registries containing detailed outcome data on miscarriage, stillbirth and congenital anomalies [26]. A similar approach could be considered for the prescription data contained within the IPCRN database.

### Clinical implications and areas for future research

Category D and X medications were considered inappropriate for prescribing during pregnancy and the results show a comparable prevalence of prescribing for these medications to previous studies and a decrease in prescription following the first visit to the GP for antenatal care. There is an opportunity to avoid unnecessary use of potentially harmful drugs in early pregnancy, through interventions targeted at increasing awareness in women of childbearing potential but also interventions to aid prescribers identify women at risk of potential teratogen use in early pregnancy, prior to the pregnancy. Risk assessments of medication use are needed due to the high prevalence of medication use in the first trimester. Arranging the initial antenatal consultation earlier could also reduce the time period in which a pregnant woman may be exposed to harmful medication.

The FDA pregnancy classification itself will be soon eliminated from product literature. Future research should also focus on the development and validation of explicit process criteria to examine the appropriateness of prescribing during pregnancy. These criteria will serve to highlight drugs to be avoided during pregnancy independent of or in the presence of certain diagnoses.

The IPCRN database can provide useful information on medicine use and prescribing practices by Irish GPs in the area of antenatal care. However, absence of data on patient morbidity and pregnancy outcomes in the current dataset limits the ability to evaluate the safety of these medicines. Future research may focus on linking IPCRN data to alternative sources of outcome data.

## Conclusion

The results of the anonymised dataset extracted by the IPCRN has provided valuable information on the current practice of antenatal prescribing within this pilot group of practices and the estimated prevalence is low by comparison internationally.

## Abbreviations

GP: General practitioner
FDA: Food and Drug Administration
IPCRN: Irish primary care research network
ATC: Anatomical therapeutic codes
GMS: General medical services
DVC: Doctor visit card
PRV: Private patient
LMP: Last menstrual period
EDD: Estimated delivery date
